# The European Lead Factory: A Blueprint for Public–Private Partnerships in Early Drug Discovery

**DOI:** 10.3389/fmed.2016.00075

**Published:** 2017-01-19

**Authors:** Anna Karawajczyk, Kristina M. Orrling, Jon S. B. de Vlieger, Ton Rijnders, Dimitrios Tzalis

**Affiliations:** ^1^Taros Chemicals GmbH & Co. KG, Dortmund, Germany; ^2^Lygature, Utrecht, Netherlands

**Keywords:** European Lead Factory, Joint European Compound Library, high-throughput screening, collaborative research, drug discovery, translational research, Innovative Medicines Initiative, public–private partnership

## Abstract

The European Lead Factory (ELF) is a public–private partnership (PPP) that provides researchers in Europe with a unique platform for translation of innovative biology and chemistry into high-quality starting points for drug discovery. It combines an exceptional collection of small molecules, high-throughput screening (HTS) infrastructure, and hit follow-up capabilities to advance research projects from both private companies and publicly funded researchers. By active interactions with the wider European life science community, ELF connects and unites bright ideas, talent, and experience from several disciplines. As a result, ELF is a unique, collaborative lead generation engine that has so far resulted in >4,500 hit compounds with a defined biological activity from 83 successfully completed HTS and hit evaluation campaigns. The PPP has also produced more than 120,000 novel innovative library compounds that complement the 327,000 compounds contributed by the participating pharmaceutical companies. Intrinsic to its setup, ELF enables breakthroughs in areas with unmet medical and societal needs, where no individual entity would be able to create a comparable impact in such a short time.

## Introduction

While we continuously obtain a better understanding of disease-causing mechanisms, we still face a number of challenges when translating these findings into therapeutic products that reach patients’ needs (1, 2).

Innovation and discoveries derived from academic research institutes have great potential to be developed into clinically meaningful products; however, it is clear that these parties often lack the resources and experience to fully progress their findings toward the clinic. Traditional pharmaceutical companies can build on decades of experience in bringing drug candidates successfully to patients. However, due to several well-reported reasons (1–3), the traditional business model faces a gap between early discoveries and product development.

Despite large investments and advances in basic and applied pharmaceutical research in recent years, the success rate in drug discovery (DD) and development of innovative therapies in most disease areas has been low as a result of this translational gap (2, 3). Consequently, the substantial need for new drugs remains and poses a serious threat not only to patients but also to the welfare of modern society (4).

In response to this necessity for new solutions, the DD landscape is continuously adjusting itself to pursue a sustainable and more productive research and discovery model (5, 6). Currently, the precompetitive space in pharmaceutical research is being redefined, and more transparent and collaborative innovation approaches are increasingly being embarked upon. The objectives are not only to share investment risks but also to minimize attrition rates of DD, pool complementary talents and resources, provide sustainable infrastructure platforms where different players have access to the brightest ideas and are able to interact liberally, and exchange collective solutions to problems arising in the DD process (7). Public–private partnerships (PPPs) are the tool of choice for bridging this gap (8, 9).

Small molecule therapy remains one of the cornerstones of modern medicine. Approximately 90% of all the therapeutics sold are based on small molecule drugs. The Food and Drug Administration’s Centre for Drug evaluation and research, which oversees the approval of new drugs, approved 45 new drugs in 2015. This marks a 19-year high after hitting a low of 18 new approved drugs in 2007 (10). Out of the new drugs approved in 2015, two-thirds are derived from small molecular entities. This exemplifies the fact that a significant number of new medicines introduced are derived from chemical compound collections.

High-throughput screening (HTS) is an effective and well-established methodology to assess the biological effect of large collections of chemical compounds. This methodology has matured over the years at large pharmaceutical companies and has generated more new active pharmaceutical ingredients than any other rational DD approach (11). However, the success of an HTS campaign highly depends on the quality of the compound collection, the infrastructure, the assays, i.e., how to read out the biological response of the screening compounds, and the data interpretation skills (12). Hence, expertise and experience from a broad range of specific life sciences must be collected and amalgamated.

With this in mind, the Innovative Medicines Initiative’s (IMI) European Lead Factory (ELF) project was launched, with an aim to create a collaborative PPP DD platform to seed and execute early-stage projects more effectively (13). By combining an industry-standard HTS infrastructure (European Screening Centre), a state-of-the-art Joint European Compound Library (JECL) and newly developed information technology (IT) solutions, ELF enables any European biotech company or research institute to access, free of charge, tools, resources, and know-how that were once available exclusively in the large pharmaceutical companies (14).

## ELF: A Collaborative Early DD Effort

**Figure 1:**
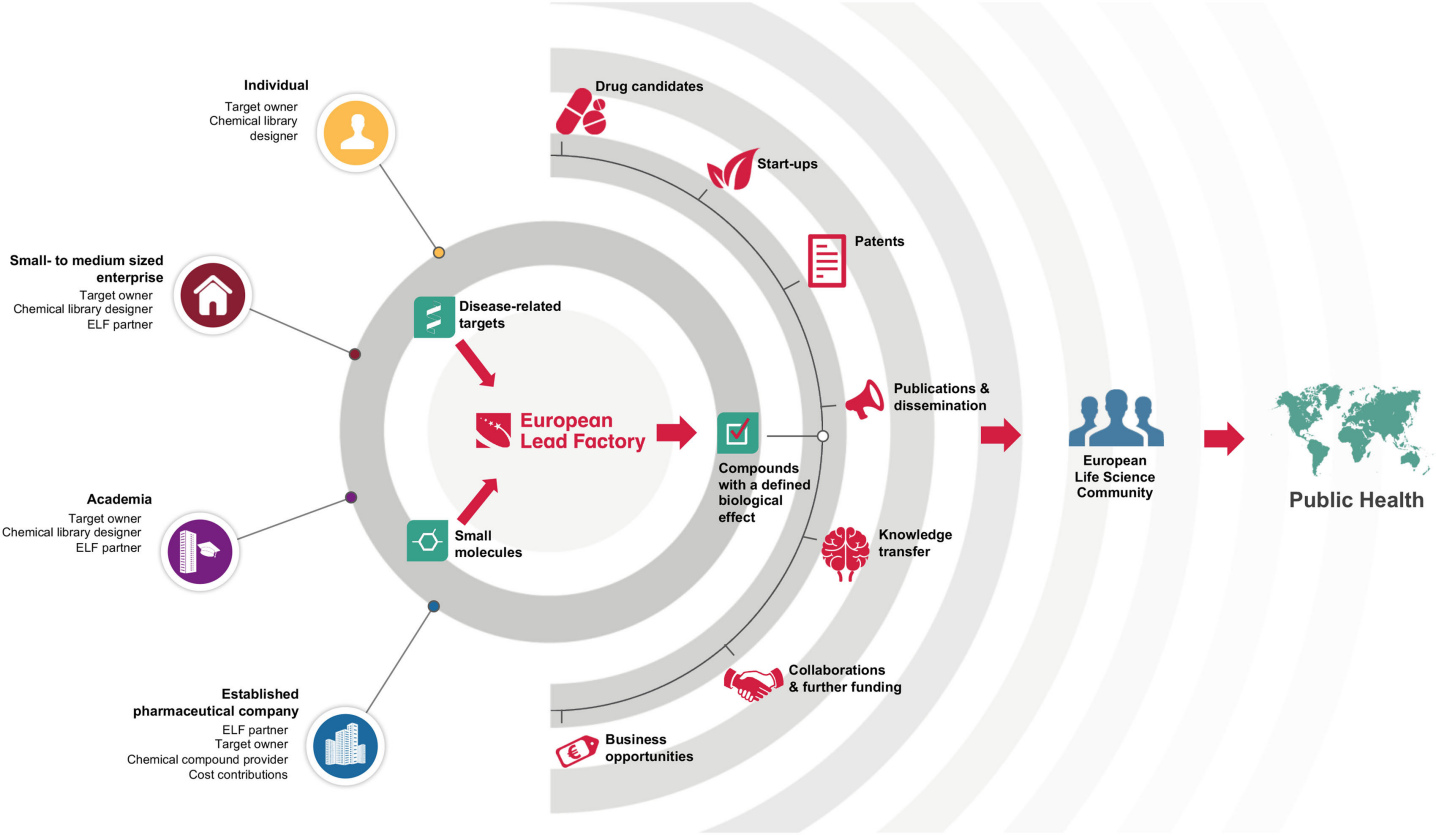
**European Lead Factory is a translational hub**. It uses the collective intelligence in the European life science community to connect disease-related targets (provided by target owners) with high-quality small molecules (based on library design ideas or provided by established pharmaceutical companies) resulting in compounds with a defined biological activity. Many types of outcomes are fed back to the community with the ultimate aim of benefiting public health.

European Lead Factory is unique in its reach, with interaction points for organizations of all sizes: from single individuals who can submit chemical library ideas and/or disease-related targets, up to the participating multinational pharmaceutical companies (Figure 1). This heterogeneity is also reflected in the core team of the ELF: it comprises 7 established pharmaceutical companies, 10 small- to medium-sized enterprises (SMEs), and 13 academic partners, who collaborate in a precompetitive mode (14). Crowdsourcing of innovative chemistry and biology allows the project to be at the forefront of these respective fields, while equipping the DD community with high-quality starting points. ELF operates as a translational hub, to which scientists from SMEs and academia, from all over Europe, can submit their ideas and have those translated into tangible assets that can be developed internally or in collaboration with partners (Figure 1). In addition, by combining innovative ideas in chemistry and biology, it opens up new areas that have yet not been explored by those currently working on DD projects.

## SMEs as an Important Driver for Success in PPPs

Public–private partnerships are, at the onset, often accused of a certain degree of inertia that results from a complex partner mix, opposing interests, democratic decision-making, and high demand of transparency. ELF has circumvented this and managed to be highly effective and fully operational within a year through a clear governance structure, intellectual property (IP) regulation, IT software solutions, in combination with the drive of the ELF partners to deliver valuable output for the benefit of the wider community. This partnership works much beyond the objectives of any single partner.

Certainly, the high ratio of SME involvement in ELF has had a pivotal role in accelerating many processes. SMEs have over the past decade worked closely with and for the larger pharmaceutical industry, as well as with public research institutes in early DD projects. ELF has provided the SMEs with a showground to further prove their abilities. The participating SMEs are instrumental in implementing the production and management of the compound library and performing the screening. Their agility and emphasis on producing results has had a professional influence on the ELF machinery, making it the well-oiled initiative it is today.

While academia and SMEs inject innovation and execution power to the PPP, the established pharmaceutical companies provide the means and experience to develop the scientific discovery of biologically active molecules into medical therapies. The extensive drug development expertise available in pharmaceutical companies provides opportunities to academics and biotechs to valorize the results either in downstream alliances along the pharmaceutical value chain, through license agreements around generated IP, or through the generation of spin-outs based on assets generated within their ELF participation.

## The ELF Mode of Action

Now fully operational, the ELF provides a unique platform for translation of innovative biology and chemistry into high-quality starting points for DD. This cohesive PPP contains three essential assets for bridging the innovation gap of early DD: (i) access to a state-of-the-art chemical compound collection (15, 16), (ii) access to screening facilities of industrial standards, and (iii) access to the expertise required to convert the obtained results and data into potential drug candidates.

## Joint European Compound Library

The quality and diversity of the compound collection is of upmost importance in DD. The JECL is one of the strongest assets of the consortium. It constitutes a collection of starting points for new therapies and consists of two parts: the pharmaceutical industry compound collection and the public compound collection. At the onset of the project, over 327,000 high-quality compounds were contributed by the seven pharmaceutical industry partners within the consortium according to exacting selection criteria agreed upon by the participating partners. Within the time frame of ELF, these compounds are being complemented with up to 200,000 newly synthesized compounds (15).

In return for their compound contributions, the participating pharmaceutical companies have access to the entire compound collection for screening a limited number of internal targets. In this way, ELF provides a neutral platform that allows different companies to access their competitors’ compound libraries. By combining screening compounds from different sources, more target classes are being addressed than by anyone single source, opening up for the revitalization of discovery projects at the participating pharmaceutical industry partner, which have failed to produce attractive hits in in-house campaigns. Consequently, the available chemical space is being utilized more efficaciously. To date, an impressive one-third of the finished ELF industry projects have triggered additional drug development efforts at the respective company. Activating such projects will eventually benefit the patients, who have an increased chance for new therapies.

Out of the 200,000 novel compounds targeted, over 100,000, based on more than 220 scaffolds (molecular frameworks), are already synthesized and available to the European Research Community midway through the project (16). More than 500 library ideas have been selected and evaluated to be processed within ELF. This new compound collection is based on proposals that are submitted by chemists—from either within or outside the consortium—using a step-by-step procedure *via* a web-based tool (17). The compound collection is unique and diverse compared to commercial sources and typical chemical compound repositories.

When submitting library proposals to ELF, external contributors retain the right to continue doing research on the chemical entities and to publish their own research and findings. Only an exclusive set of compounds that is being synthesized within ELF is being kept proprietary. In order to ensure the quality of the produced library, submitted library proposals are being assessed by a Library Selection Committee. It consists of eight members from the pharmaceutical industry, SMEs, and academia, all bound by confidentiality, in order to provide broad and complementary chemistry and DD expertise, thereby ensuring a high-quality library. The data and information provided at a library proposal stage are used to assess the original proposals against six specific drug relevant selection criteria: novelty, molecular properties, synthetic tractability, diversity potential, structural features, and innovative library design. Furthermore, molecular properties and structural features should be preferably aligned to contemporary hit- and lead-like properties (15).

Given the size and heterogeneity of the chemistry partners within the ELF consortium (23 out of the 30 partners are involved in the chemistry activities), chemistry project management is critical to its success. Real-time monitoring, data storage, and decision-making are enabled by TarosGate 2, a secure informatics platform, which seamlessly connects the chemistry laboratories of the academic and SME partners. Since the software covers three important aspects of the decentralized collaborative work: (i) work documentation as an electronical lab journal, (ii) management of the project, and (iii) communication, it has a noticeable impact on the operational speed of the chemistry consortium.

## European Screening Center (ESC)

One of the greatest challenges in the postgenomic era is how to translate the host of genetic knowledge into drug targets, i.e., proteins or biological structures, which can be modulated to give a desired biological effect. The number of available targets is greater than ever. To dissect which ones are worth pursuing has proven difficult and associated with a great need of highly interdisciplinary collaborations. A researcher at any European SME or academic institute is welcome to submit a proposal to ELF for screening his or her drug target. The proposals are reviewed based on scientific value and technical maturity. The last is defined by the requirements for the screening assay, which has to be compatible with HTS and executable in well plates, together with the availability of complementary assays to further refine the hit compound selection.

The drug targets selected from the European life science community are being screened at the ESC. Once the assay is transferred, the work begins by optimizing it further. After a first screen against all available compounds in the collection (currently over 400,000), the so-called primary hits (typically a few thousands) are further evaluated to confirm engagement with the defined drug target and to establish activity levels of desired and undesired biological effects. At this point, three to five different types of assays have been applied and the number of interesting compounds is narrowed down to typically a few hundreds. The ELF medicinal chemists make the final selection of the best ≤50 compounds, based on information provided by the researcher who proposed the target and the purpose of the research (drugs vs. tool compounds) (12).

As of end of August 2016, 72 public target projects have been selected by ELF. In order to extract the most interesting compounds for these projects, more than a total of 150 biochemical, cellular, and biophysical assays have been developed, 49 HTS have been completed and over 1,000 compounds have been granted to owners of public target projects that can serve as potential new starting points for DD projects. Further work to validate the selected compounds have been performed for 23 of the 30 projects. In this process, >1,500 bespoke compounds have been synthesized by the ELF medicinal chemists and provided to their target owners.

**Figure 2:**
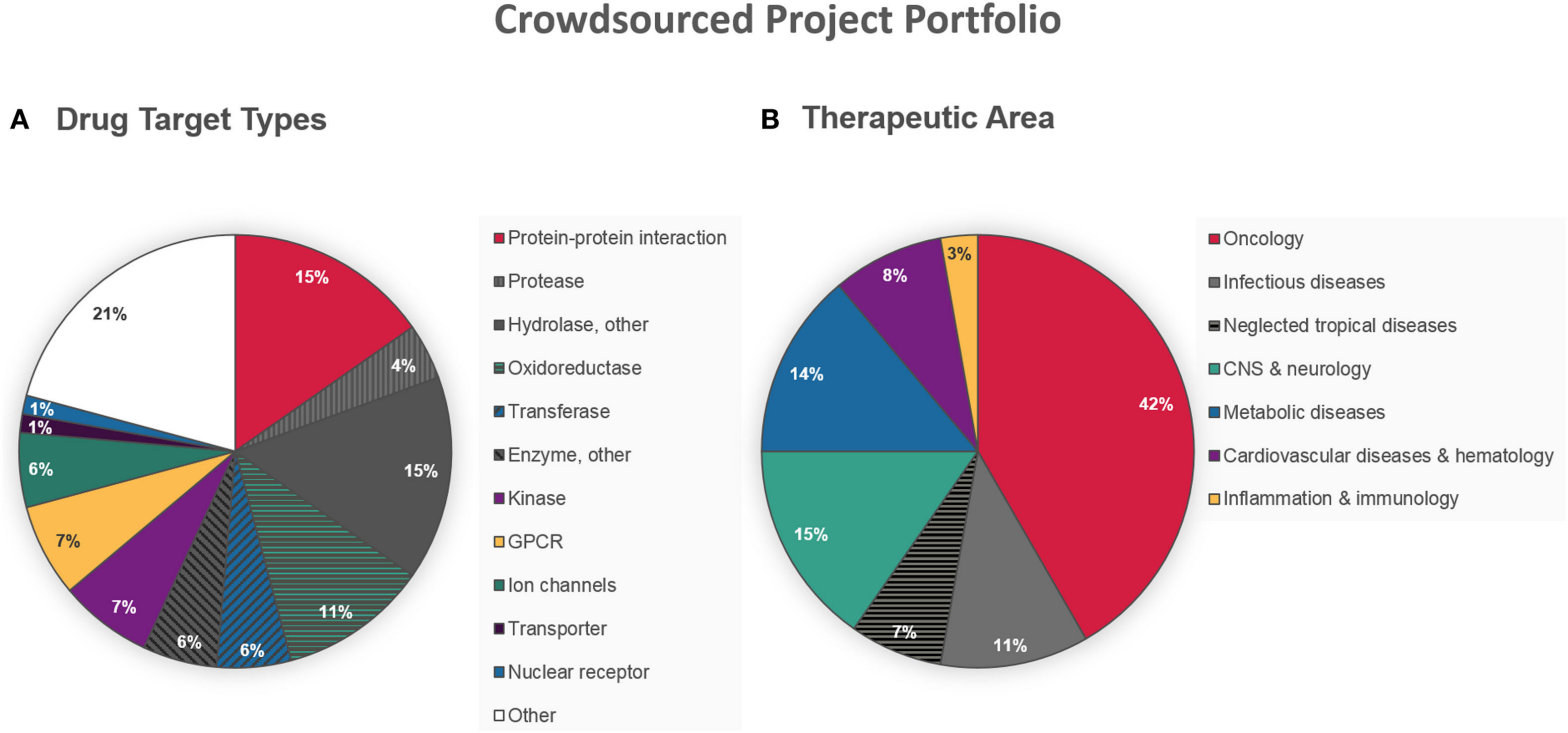
**Character of crowdsourced disease-related target projects accepted by European Lead Factory (ELF) as of August 31, 2016**. **(A)** Nature of molecular drug target. **(B)** Main therapeutic area addressed by the projects.

From the very start, ELF aimed for non-discriminating crowdsourcing activity with a special focus on innovation. As a consequence, the current drug target project portfolio is complementary and clearly different from the industry (13, 18). It includes many targets considered as highly challenging, such as protein–protein or protein–DNA/RNA interactions (15% of the projects), and very few of the most pursued target types, e.g., kinases (7%) and proteases (4%) (Figure 2A). Interestingly, the stringent requirement for an HTS-compatible assay format does not seem to have affected the innovative aspect on target level. Although the projects are spread over a wide range of disease areas, illustrated in Figure 2B, there is a high proportion of oncology projects (42%). This might be a reflection of the relatively abundant funding opportunities for cancer research, leading to a higher chance of accessing the infrastructure needed to develop an HTS-compatible assay. The aspiration for societal benefit is illustrated by the number of projects in infectious diseases (18% in total). In fact, ELF recently lowered the hurdles for non-profit DD projects in the area of neglected tropical diseases, freeing charities, and other organizations from financial obligations in their pursuit of new therapies for patients in the least developed countries (18).

It is important to emphasize that the control and IP rights of the biology drug target projects remain with the original proposer. This secures the involvement of the biology experts within the ELF to efficiently use the collective intelligence in translating the findings into clinically relevant small molecules. A fit-for-purpose IP framework allows successful projects to be further progressed to clinical studies, while research use of results is facilitated. ELF projects derived from academia and SMEs choosing to look for a commercial partner have to provide the ELF pharmaceutical industry partners with a right of first option, which the target owner have the right to refuse. For the participating industry partners, this provides an additional opportunity to have insight into novel innovative drug targets that these pharmaceutical companies would not have access to. The Honest Data Broker is a consortium software developed specifically for ELF to balance the scientific and IP requirements of all the involved parties. It ensures that all the partners work together in a productive, and where required, transparent environment, as one single unit, despite the different nature and interests of the various participating stakeholders (academia—SME—pharmaceutical industry) (19).

## Conclusion

Three years after the start of the project, ELF has matured from a start-up initiative to a well-organized group of over 150 scientists. Experts from all essential areas of early DD—industry, academia, and SMEs—now jointly produce high-quality output for the wider public benefit. In this time, the PPP has published >30 articles and the results from the first crowdsourced ideas gradually disseminate in the public domain for everyone to assess (6, 12, 13, 15, 19–48).

At the time of writing this paper, researchers from 13 countries are involved, one spin-out company has been established, and patents have been filed by academic research groups based on the screening results of their submitted screening proposals to the ELF. Over 120,000 new compounds have been synthesized and added to the screening library. So far, 72 screening programs have been accepted from European academic groups and biotechs and are currently being processed.

The resulting biologically active compounds are used by biotechs to further progress their DD programs, by universities to be optimized toward potential therapies, or used as chemical tools to support groundbreaking basic research. For the pharmaceutical industry partners, currently 17 out of 49 screening campaigns with the ELF compound library have triggered further work within the companies.

A less tangible, but just as important, outcome of this PPP is the impact it has on the human capital involved. The constant flow of knowledge exchange between scientists in different disciplines and organizations inside and outside ELF allows to fully capitalize on the collective intelligence.

## Outlook

Unmet medical needs still prevail in nearly all indication areas. Although several initiatives have been launched in the past few years on a regional, national, or international scale (49–53), ELF is the first effectively operating pan-European initiative embracing both the public and private sectors.

European Lead Factory provides a unique platform for connecting innovative biology and novel chemistry into high-quality starting points for DD. Intrinsic to its setup, ELF enables breakthroughs in areas with unmet medical needs, where no individual entity would be able to create a comparable impact in such a short time.

With an active life science community in Europe, the results will be absorbed and further developed into clinically meaningful drug candidates. While having bridged a major gap in developing innovation in chemistry and biology toward an interesting DD project, it is already clear that the next translational gap is already emerging. Valuable compounds derived from JECL have now been extensively tested *in vitro* and have seen some preclinical *in vivo* models, but the subsequent development of those compounds into drug candidates needs the next push. Currently, some of the DD projects flow into other initiatives within the IMI portfolio, or are under evaluation by other funding organizations such as the Wellcome Trust or pharmaceutical companies to be further developed. Facilitating such a subsequent step will be an important driver to increase the long-term impact of ELF on the European community. Together with the essential stakeholders for the next phase, these types of PPPs can jointly shape the future of medicine. In fact, ELF could serve as a blueprint of how future PPPs might operate in order to efficiently find cures that could reach patients in dire need of new treatments.

## Author Contributions

AK, KO, JV, TR, and DT contributed equally to the writing and the editing of the manuscript.

## Disclaimer

All the authors are members of the European Lead Factory consortium.

## Conflict of Interest Statement

The authors declare that the research was conducted in the absence of any commercial or financial relationships that could be construed as a potential conflict of interest.

## Abbreviations

DD, drug discovery; ELF, European Lead Factory; ESC, European Screening Center; FDA, Food and Drug Administration; HDB, honest data broker; HTS, high-throughput screening; IMI, Innovative Medicines Initiative; IP, intellectual property; IT, information technology; JECL, Joint European Compound Library; NTDs, neglected tropical diseases; PPP, public–private partnership; SME, small- to medium-sized enterprise.
